# Digital health interventions to promote healthy lifestyle in hemodialysis patients: an interventional pilot study

**DOI:** 10.1038/s41598-024-53259-x

**Published:** 2024-02-03

**Authors:** Wen-Yi Li, Jiang-Chou Yeh, Cheng-Chih Cheng, Su-Hua Huang, Hui-Chin Yeh, Bor-Wen Cheng, Jou-Wei Lin, Feng-Jung Yang

**Affiliations:** 1 Renal Division, Department of Internal Medicine, National Taiwan University Hospital Yun Lin Branch, No. 579, Sec. 2, Yunlin Rd., Douliu, Yunlin County 640 Taiwan; 2 College of Medicine, National Taiwan University, Taipei, Taiwan; 3 Department of Industrial Engineering and Management, National Yunlin University of Science and Technology, Douliu, Taiwan; 4 Department of Dietetics, National Taiwan University Hospital Yun Lin Branch, Douliu, Taiwan; 5 Department of Applied Foreign Languages, National Yunlin University of Science and Technology, Douliu, Taiwan; 6 Cardiovascular Division, Department of Internal Medicine, National Taiwan University Hospital Yun Lin Branch, Douliu, Taiwan

**Keywords:** Kidney, Quality of life, Rehabilitation

## Abstract

Low physical activity has been associated with poor prognosis in hemodialysis (HD) patients. Interventions to maintain healthy lifestyle in this population are important to reduce mortality. This study aimed to evaluate the effectiveness of digital health interventions (DHIs) for improving the physical activity and health-related quality of life (HRQoL) in HD patients. The 24-week prospective study enrolled 31 clinically stable HD patients. All participants were assigned home exercises and provided with wearable devices. Dietary and exercise information was uploaded to a health management platform. Suggestions about diet and exercise were provided, and a social media group was created. Physical performance testing was performed at baseline and during weeks 4, 8, 12, 16 and 24. HRQoL and nutritional status were evaluated. A total of 25 participants completed the study. After the interventions, the daily step count increased 1658 steps. The 10-time-repeated sit-to-stand test reduced by 4.4 s, the sit-to-stand transfers in 60 s increased 12 repetitions, the distance of six-minute walk test (6MWT) increased by 55.4 m. The mental health components and burden of kidney disease of the Kidney Disease Quality of Life survey, and subjective global assessment (SGA) scores improved. By Spearman correlation, the monthly step count correlated positively with 6MWT and SGA. DHIs that combined wearable devices, a health management platform, and social media could strengthen physical activity and improve the HRQoL and nutrition of maintenance HD patients. The results outline a new model to promote healthy lifestyle behaviors in HD patients.

## Introduction

End-stage renal disease (ESRD) has been increasing in recent decades. In Taiwan, the incidence and prevalence of ESRD is among the highest in the world^1^. The progression of chronic kidney disease (CKD) has an impact on the decrease in functional capacity in this vulnerable patient group^2^. People with ESRD often have other comorbidities such as hypertension, diabetes, and cardiovascular disease. Lifestyle modifications, including sufficient physical activity and adequate diet control are important for these chronic illnesses^3^. Low physical activity has been associated with poor prognosis in ESRD patients on hemodialysis (HD)^4,5^. Reductions in physical activity are significantly associated with poor prognosis and are independent of baseline physical activity^6^. Most HD patients are physically inactive, particularly on days when they undergo HD treatment^7^.

Engaging in physical activity is associated with decreased mortality risk among HD patients. The benefits of exercise in dialysis patients are well established, and support the prescription of physical activity in regular treatment^8^. Interventions to maintain physical activity in this vulnerable population are important. The Renal Exercise Demonstration Project in the United States showed that an initial lower physical component scale, on the Medical Outcomes Study Short-Form 36 questionnaire, could benefit more from exercise counseling in both objective measures and self-reported physical functioning^9^. A Japanese prospective cohort study found a substantial mortality benefit among HD patients whose physical activity comprised at least 4000 steps per non-dialysis day^10^. According to National Institute for Health and Care Excellence (NICE) clinical practice guidelines, physical activity and exercise should be encouraged in the HD population where there were no contraindications^11^. There was no consensus about the types and intensity of physical activity in HD patients. In the previous investigations, exercise sessions were mostly scheduled during HD (> 60%), and the training program was prescribed three times a week^12^. One recent meta-analysis revealed home-based exercise programs were beneficial to CKD stage 3–5 patients in health-related quality of life (HRQoL), functional capacity and symptoms of depression^13^.

Digital health interventions (DHIs) notably manage chronic conditions to improve health literacy, self-efficacy and health-related behaviors such as physical activity, diet control and adherence^14^. A health management platform was used widely in telehealth interventions^15^. The apps—application software programs that run on mobile devices—are also a recent innovation for delivering health information and education to patients^16^. Our previous randomized control study applied wearable devices, a health management platform, and social media support for 12 weeks to patients with CKD stage 1–4. The intervention group had significantly higher levels of self-efficacy and self-management. HRQoL and the daily step count increased in the intervention group. The decline in estimated glomerular filtration rate (eGFR) was significantly slower in the intervention group. These results outlined a new self-management model to promote healthy lifestyle behaviors in CKD patients^17^. However, there have been limited studies evaluating the effects of health promotion using wearable devices, health management platforms and social media in HD patients. Thus, we investigated about DHIs in improving participants’ physical activity and HRQoL.

## Methods

### Study design

This study was a trial utilizing pretest–posttest design. The study protocol was approved by the Research Ethics Committee of National Taiwan University Hospital (No. 201812145RINA). The ClinicalTrials.gov number was NCT05281497]. Written informed consent was obtained from all participants before starting the study. All methods were performed in accordance with the relevant guidelines and regulations (the Declaration of Helsinki). The first trial registration was on 01/07/2019.

### Study population

This study prospectively enrolled ESRD patients undergoing HD from the HD center of National Taiwan University Hospital (NTUH) Yunlin branch and Ming-De HD clinic between July 2019 and December 2019. The NTUH Yunlin branch is a regional teaching hospital located in a suburban area in mid-Taiwan. The Ming-De HD clinic is a HD clinic with 41 beds for stable HD patients. The patients underwent thrice-weekly standard bicarbonate HD, with a target Kt/V of at least 1.2 using single-use dialyzers with high-efficiency or high-flux membranes^18^. Patient inclusion criteria were aged 20 years and above with a diagnosis of ESRD and having received HD maintenance for more than 3 months. Those who agreed to participate in the study signed informed consent forms. Exclusion criteria were an inability to use a smartphone, impaired walking capacity or a psychiatric disorder, or any hospitalization during the previous 3 months. If the participants changed dialysis modality or received renal transplantation during the study period, they would be excluded because of incomplete interventions.

### Data collection

All participants undertook a brief interview to document their demographic profiles. The diagnosis of comorbidity was documented by clinically relevant histories or medical examinations. Because HD patients had high cardiovascular risk and reduced exercise tolerance, a Physical Activity Readiness Questionnaire (PAR-Q)^19^ was performed before the intervention. PAR-Q offered a safe preliminary screening of candidates for exercise testing and prescription. Laboratory data including hemograms and serum biochemistry, were measured as per the care routine for ESRD patients according to the guidelines of the Taiwan Society of Nephrology. All blood samples were drawn before dialysis before the first HD session in the week.

### Description of the intervention

**Figure 1:**
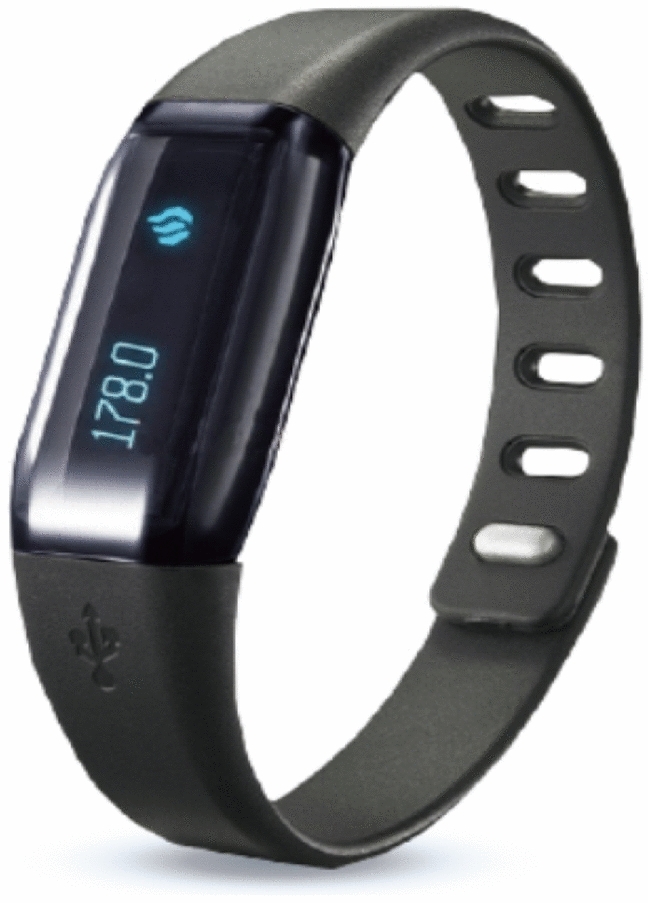
Heart Rate Smart Wristband, GSH405-B6, Golden Smart Home Technology Corporation.

The researchers included a nephrologist and her assistants, who were from graduate school of health industry management in National Yunlin University of Science and Technology. They were also supervised by physical therapists and dietitians. Each participant was provided with a wearable device (a Heart Rate Smart Wristband, GSH405-B6, Golden Smart Home Technology Corporation) (Fig. 1). The wristband was approved by the National Communications Commission of Taiwan (NCC verification code: CCAB16LP1430T3). The device could detect steps (0–120,000 steps, division 1 step), calories, and sleep periods. The wearable device was validated in previous studies^17,20^.

**Figure 2:**
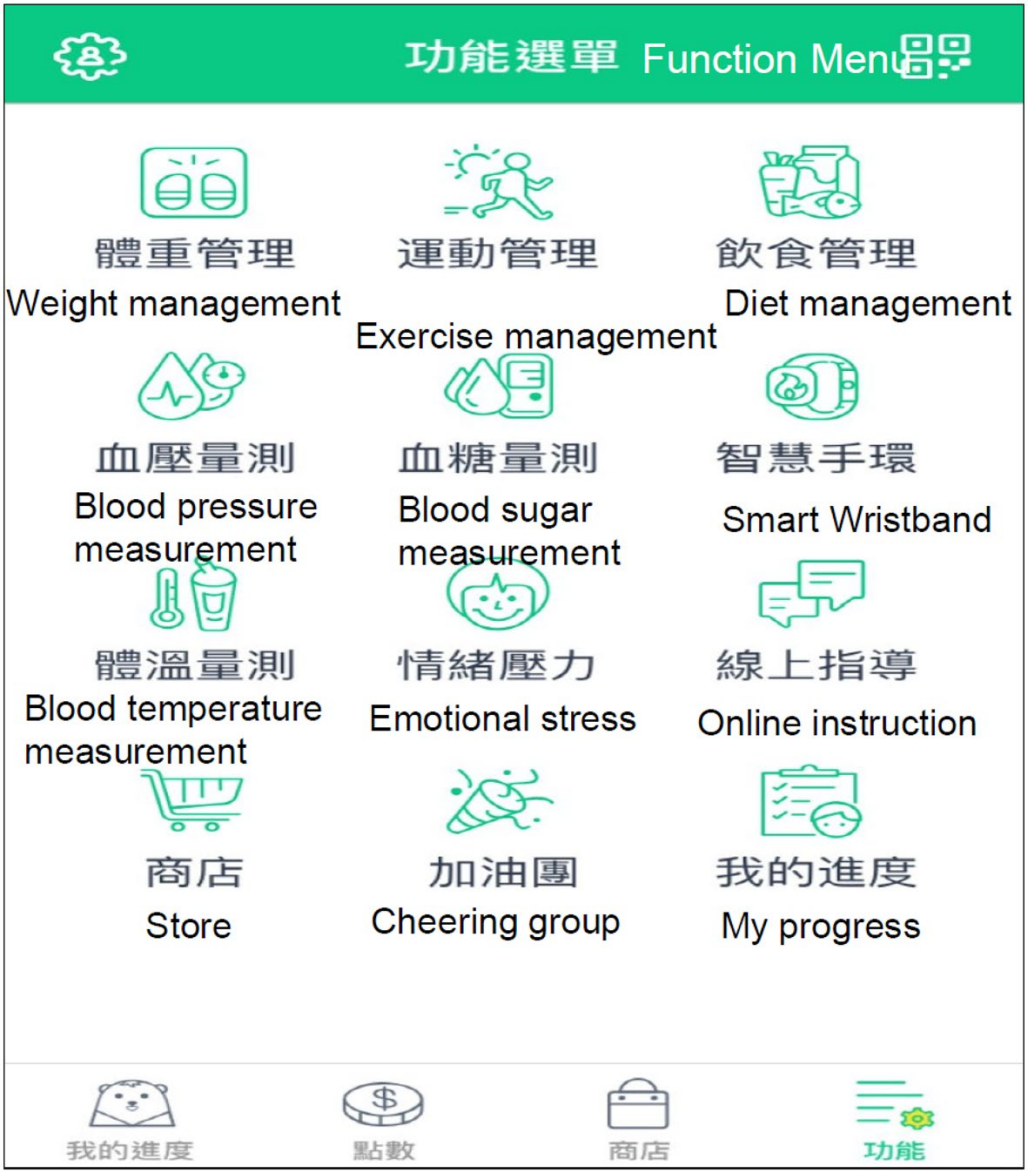
User interface of the health management platform software **(**WowGoHealth App).

Each participant downloaded an app (WowGoHealth app) (Fig. 2) to connect with the health management platform (GSH AI health platform). Participants’ exercise-related data, including number of steps walked, distance, consumed calories, and heart rate, were collected through the wearable devices. All participants were taught to record a dietary diary by taking photos of meals and using a smartphone application. All collected information was uploaded to the health management platform. Only the researchers could access the data on the health management platform. The researchers analyzed the pictures every day with average 3–5 pictures to calculate the calories and nutrients in the food, such as starches, proteins, and lipids, and the percentage of vegetables and fruits. The researchers made suggestions about diet to the participants.

**Figure 3:**
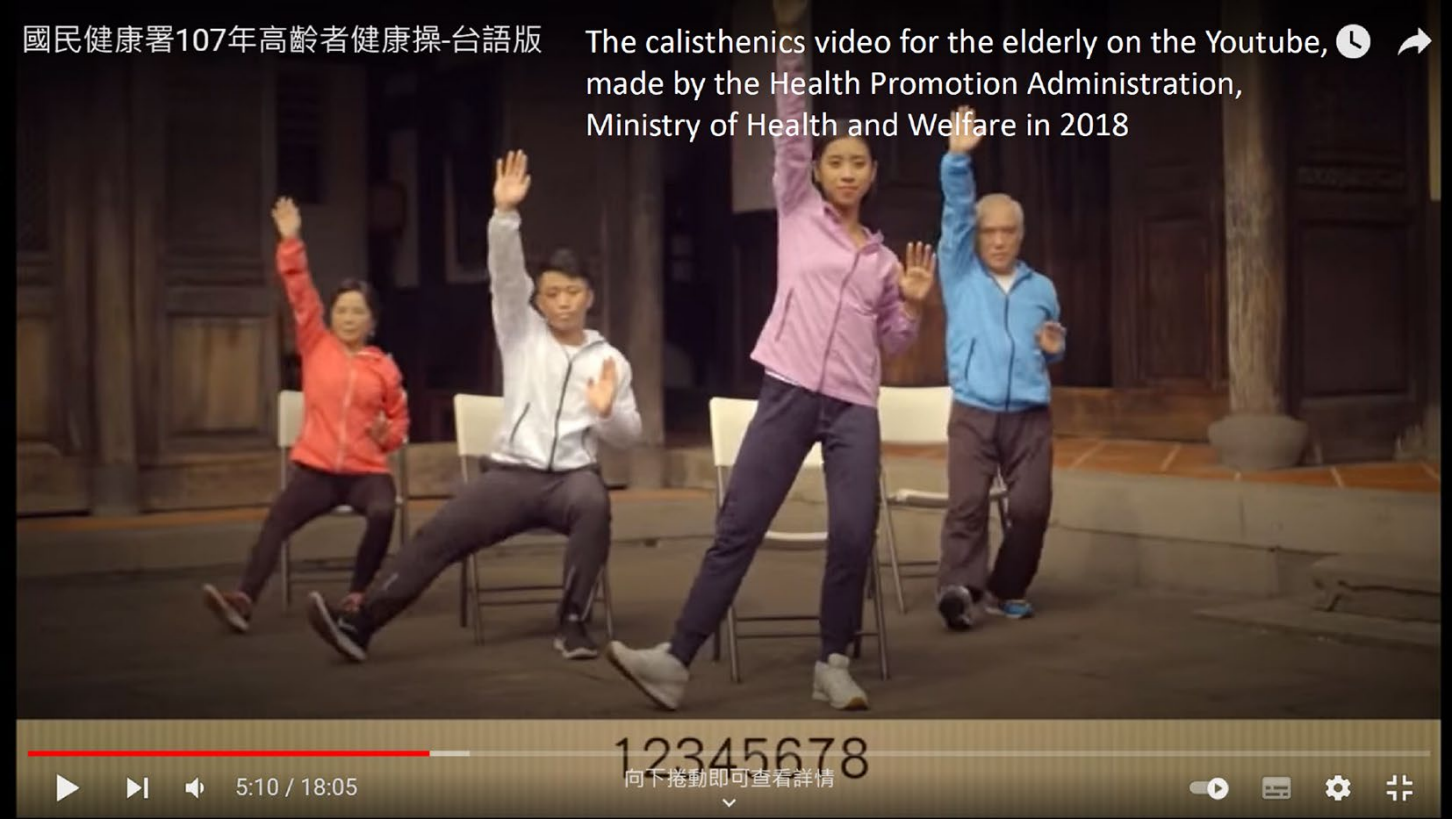
An 18-min calisthenics video made by the Health Promotion Administration, Ministry of Health and Welfare Mandarin Version: https://youtu.be/_w50TfdCmKU; Hokkien Version: https://youtu.be/-Otr4BlYm2E (Adapted from the official website: https://youtube.com/user/hpagov).

Exercise included an 18-min calisthenics program made by the Health Promotion Administration, Ministry of Health and Welfare (Fig. 3). The video included stretching, aerobic and resistance training, and could be performed in a sitting or standing position. Participants were asked to perform the calisthenics at least three times a week. A daily step count of 7500 steps was set according to a prospective cohort study which revealed that mortality rates progressively decreased before leveling off at approximately 7500 steps per day^21^.

LINE is a mobile app operated by the LINE Corporation. All users can use texts, images, videos, and audio for communication at any time. In Taiwan, LINE is the predominant text messaging app. A LINE group was created to inspire the participants, especially when the daily target step count had been reached. Teleconsultations of health information were provided via LINE. All the interventions lasted for 24 weeks.

### Outcome measurement instruments

All outcome measures were collected at baseline, and weeks 4, 8, 12, 16 and 24 after initiation of the study.

### Primary outcomes

Sit-to-stand-10 (STS-10) tests measured the time taken to complete 10 sit-to-stand cycles. Participants were instructed to start and finish the test in a seated position on a standard chair which was positioned against a wall. Patients stood from a seated position and sat back down again as quickly as possible, with their arms folded across their chest. The time taken to perform 10 repetitions was recorded^22^. Sit-to-stand-60 (STS-60) measured the number of repetitions of sit-to-stand cycles achieved in 60 s^23^. The STS-10 and STS-60 were suitable for measuring lower extremity muscle strength and had high test–retest reliability in ESRD patients^23^.

The 6-min walk test (6MWT) was used as an index of exercise capacity. The 6MWT was performed according to the statements of the American Thoracic Society^24^. Participants were instructed to walk as fast as they could for 6 min on a flat 30-m track. They were allowed to stop and rest during the test but were instructed to resume walking as soon as they felt able to do so. The distance walked was recorded^23^.

**Figure 4:**
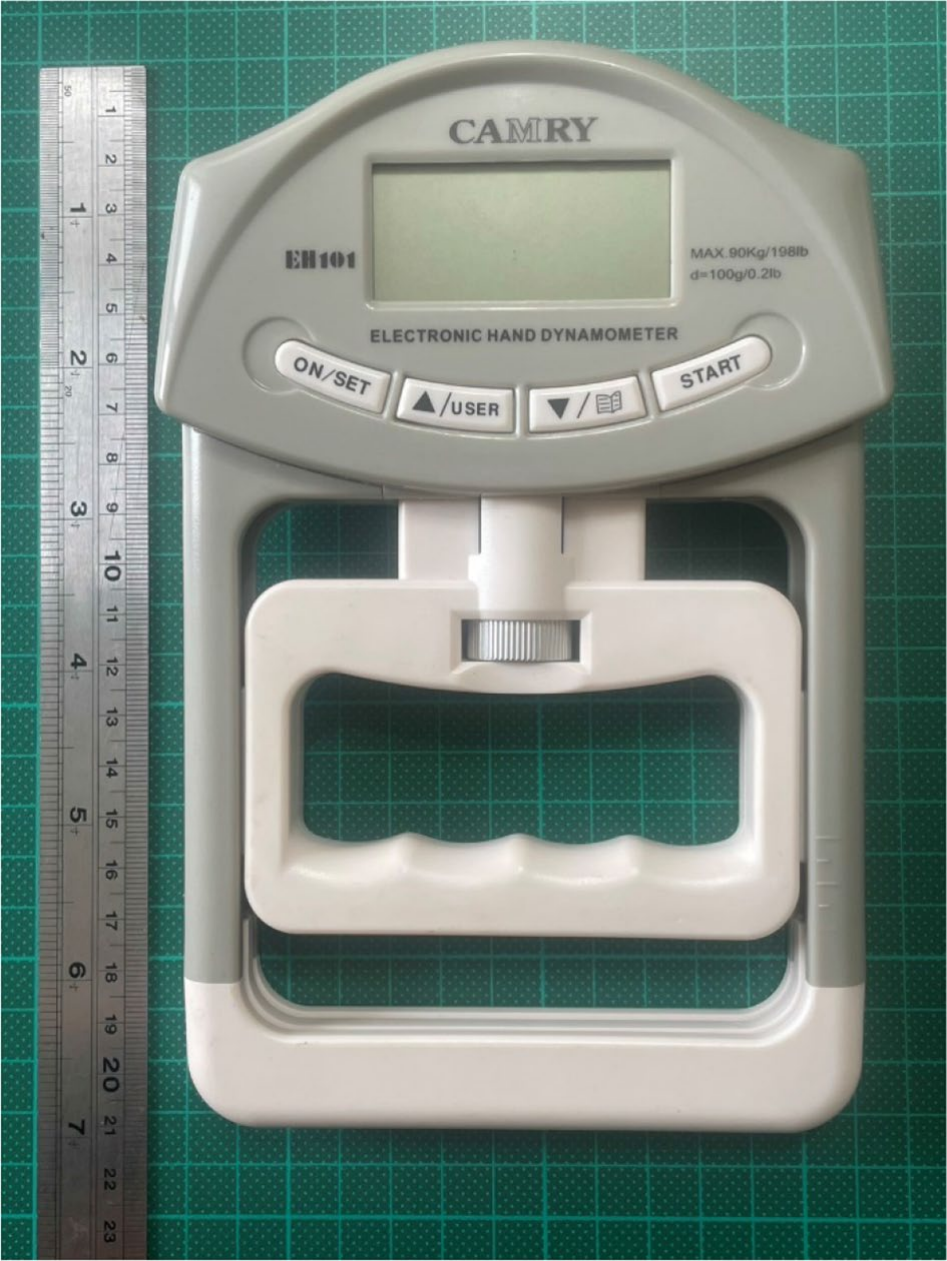
CAMRY digital hand dynamometer EH101 was used to measure the hand grip strength developed by each hand.

Hand grip strength (HGS) was an easily performed test and correlated well with lean body mass in ESRD patients^25^. It was an independent outcome predictor of male ESRD patients^26^. A handgrip dynamometer (CAMRY digital hand dynamometer EH101, Fig. 4) was used to measure the amount of strength developed by each hand. The device could capture the electronic hand grip automatically. It provided excellent reliability and validity as Jamar dynamometer, which was a widely recognized tool for measuring grip strength^27^. The participants performed STS-10, STS-60, 6MWT and HGS before the third HD session of the week.

### Secondary outcomes

HRQoL was measured by the Kidney Disease Quality of Life survey (KDQOL-36™)^28^. The KDQOL-36™ was a short form that includes the 12-Item Short Form Survey (SF-12) as a generic core plus the burden of kidney disease, symptoms/problems of kidney disease, and effects of kidney disease scales from the KDQOL-SF™ v1.3. Items 1–12 were SF-12; items 13–16 were burden of kidney disease; items 17–28 were symptoms/problems; while items 29–36 were effects of kidney disease. The higher the scores, the better the HRQoL. The Cronbach’s alpha was estimated in excess of 0.8^28^.

The Subjective Global Assessment (SGA) was a reliable tool for the evaluation of nutritional status and for detection of protein-energy wasting (PEW) in dialysis patients^29^. The 7-point SGA scored according to the patients’ history of weight change in the previous 6 months, dietary intake, and presence of gastro-intestinal symptoms (loss of appetite, nausea, vomiting, and diarrhea). A physical examination of loss of subcutaneous fat mass and muscle wasting was conducted. A score of 6–7 indicated a normal nutritional status, a score of 3–5 indicated mild to moderate PEW, and a score of 1–2 indicated severe PEW^30^.

### Statistical analysis

Statistical analyses were performed using IBM SPSS Statistics for Windows, version 22.0.0 (IBM Corporation), and a 2-sided *P* value < 0.05 was considered significant. We examined the distribution of all outcome data. The distributional properties of the data were expressed as mean ± standard deviation for continuous variables with a normal distribution or median (interquartile range) for those with a skewed distribution. The repeated measures ANOVA was used to compare the outcomes, and F test with Greenhouse–Geisser adjustment (F-GG) was used to correct the degrees of freedom of the *F*-distribution^31^. For categorical variables with percentages (%), a chi-square or Fisher’s exact test was used. Pearson or Spearman correlation (normal or non-normal distribution, respectively) was used for analysis of association of monthly step count with the physical function, HRQoL, SGA, and laboratory data.

### Ethics approval and consent to participate

The study is approved by National Taiwan University Hospital’s Research Ethics Committee (No. 201812145RINA), and informed consent was acquired from all participants. The ClinicalTrials.gov number was NCT05281497. The first trial registration was on 01/07/2019.

## Results

### Baseline participant characteristics

**Figure 5:**
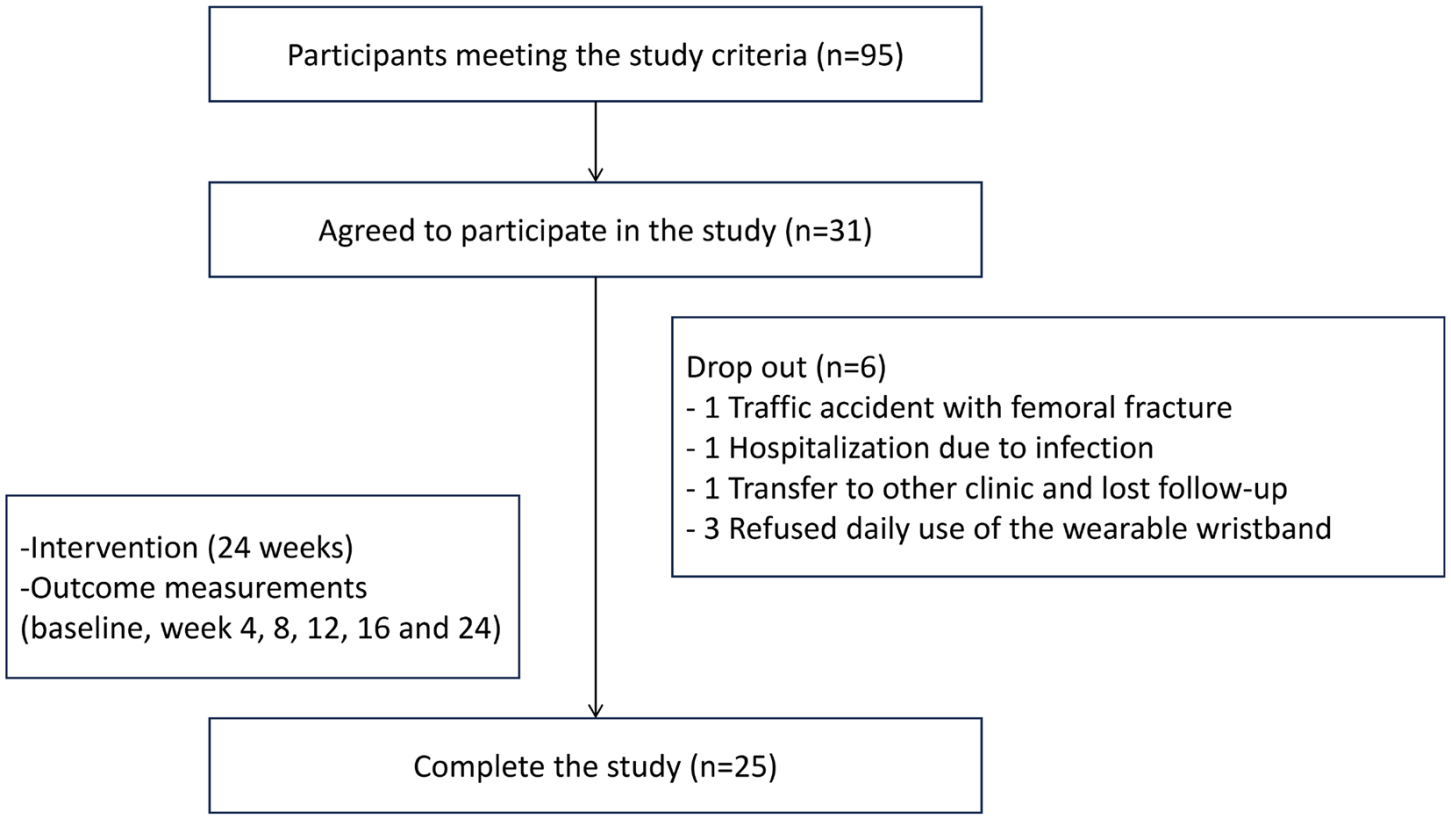
Flow chart of this study.

Thirty-one participants completed the pretests. Six patients withdrew from the study (Fig. 5). A total of 25 participants completed the posttests. There were no dropouts caused by complications related to the intervention. The mean age of all participants was 54.0 years (standard deviation [SD] 12.9), and 56% were male. The baseline characteristics of these participants are presented in Table 1. The HD duration was 6.0 ± 6.1 years. Most of the participants had a senior high school degree (n = 11, 44%). Hypertension was the most prevalent comorbidity (96%), and 36% of participants had diabetes mellitus. Their body mass index was 23.6 ± 3.9 kg/m^2^. Their baseline serum albumin and hemoglobin levels were within the normal range.

**Table 1:** Baseline characteristics of the participants (n = 25).

	All (n = 25)
Men, n (%)	14 (56)
Age, years	54.0 ± 12.9
≥ 65, n (%)	5 (20)
HD duration (years)	6.0 ± 6.1
Level of education, n (%)	
Elementary school	2 (8)
Junior high school	4 (16)
Senior high school	11 (44)
College or university	5 (20)
Masters degree	3 (12)
Comorbidities, n (%)	
Diabetes mellitus	9 (36)
Hypertension	24 (96)
Dyslipidemia	9 (36)
Coronary artery disease	8 (32)
Malignancy	2 (8)
Body mass index, kg/m^2^	23.6 ± 3.9
Laboratory parameters	
Hemoglobin, g/dL	11.0 ± 1.2
Creatinine, mg/dL	12.2 ± 2.9
Albumin, g/dL	4.1 ± 0.3

*HD* hemodialysis, *eGFR* estimated glomerular filtration rate.

### Primary outcomes

#### Physical function (Table 2)

**Table 2:** Summary of primary outcome measures and monthly step count.

Variables	Baseline	Week 4	Week 8	Week 12	Week 16	Week 24	F	*P *value	Post hoc comparison
	Mean (SD)	Mean (SD)	Mean (SD)	Mean (SD)	Mean (SD)	Mean (SD)			
Steps per month	153,120 (69,635)	209,988 (115,986)	212,037 (120,572)	196,642 (94,785)	189,207 (82,951)	199,557 (85,323)	11.854	0.000***	Baseline < Week 4, 8, 16 and 24 Week 16 < Week 8 and 24
Steps per day	5468.6 (2487.0)	7499.6 (4142.4)	7572.8 (4.306.1)	7022.9 (3385.2)	6757.4 (2962.5)	7127.0 (3047.3)			
STS-10 (second)	19.0 (5.6)	14.5 (3.9)	13.6 (3.8)	14.3 (5.2)	14.5 (5.3)	14.6 (5.4)	13.416	0.000***	Baseline > Week 4, 8, 16 and 24
STS-60 (time)	30.2 (9.9)	39.3 (10.5)	43.4 (12.5)	42.4 (11.3)	42.0 (11.3)	42.2 (10.7)	20.716	0.000***	Baseline < Week 4 < Week 8 Baseline < Week 16 and 24
6MWT (meter)	413.4 (65.8)	455.6 (70.6)	485.0 (73.4)	470.1 (73.6)	466.4 (69.0)	468.8 (68.6)	12.921	0.000***	Baseline < Week 4 < Week 8 Baseline < Week 16 and 24
HGS, non-dominant hand (kg)	25.4 (11.2)	26.4 (10.8)	26.4 (9.3)	27.5 (11.6)	27.7 (11.6)	27.6 (11.6)	2.787	.054	
HGS, dominant hand (kg)	27.0 (10.6)	28.5 (10.9)	28.1 (10.2)	28.9 (11.8)	28.7 (11.8)	28.9 (11.9)	2.651	.070	

*STS-10* sit-to-stand 10, *STS-60* sit-to-stand 60, *6MWT* 6-m walk test, *HGS* hand grip strength.

*P < 0.05, **P < 0.01, *** *P* < 0.001.

Monthly step counts were non-normally distributed, and the median of baseline monthly steps were 123,115 (interquartile range 102,458–181,744, average daily steps 4397). Monthly step counts increased from 153,120 ± 61,635 steps (daily steps counts 5468.6 ± 2201.3 steps) to 209,988 ± 115,986 steps (daily steps counts 7499.6 ± 4142.4 steps) within the first 4 weeks (*P* < 0.001). The monthly step-counts reached a peak in week 8 (212,037 ± 120,572) with an average of 7572.8 steps per day. By the end of the study, the monthly step count had increased by 46,437 steps compared with the baseline (*P* < 0.001), by an average increase of 1658 steps per day.

The baseline STS-10 was 19.0 ± 5.6 s, and by week 4 was 14.5 ± 3.9 s, which represented much improvement (*P* < 0.001). At the end of the study, the STS-10 measurement was 4.4 s shorter than the baseline (*P* < 0.001).

The baseline STS-60 was 30.2 ± 9.9 times, and the STS-60 at week 4 was 39.3 ± 10.5 times, which represented a significant improvement (*P* < 0.001). The STS-60 at week 8 was 43.4 ± 12.5 times. This change was also significant compared with the STS-60 result from week 4 (*P* = 0.002). The values of STS-60 at week 12, 16 and 24 were similar. At the end of the study, the STS-60 measurement increased 12 times relative to the baseline (*P* < 0.001).

The baseline 6MWT was 413.4 ± 65.8 m, and the 6MWT at week 4 was 455.6 ± 70.6 m, which demonstrated an improvement (*P* < 0.001). The 6MWT at week 8 reached a peak value (485.0 ± 73.4 m, *P* = 0.003). The 6MWT at week 12, 16 and 24 were similar, but better than the baseline data. At the end of the study, the 6MWT increased by 55.4 m compared with the baseline (*P* < 0.001).

The baseline HGS was 25.4 ± 11.2 kg for the non-dominant hand, and 27.0 ± 10.6 kg for the dominant hand. The baseline HGS of non-dominant hand was smaller than the HGS at week 12, 16 and 24. The baseline HGS of dominant hand was smaller than the HGS at week 4, 12, 16 and 24. Compared with baseline data, the non-dominant hand and dominant hand HGS had increased by 2.2 kg and 1.9 kg, respectively, without statistical significance (*P* = 0.054, and *P* = 0.070, respectively) by the end of study.

### Secondary outcomes

#### Health-related quality of life and nutrition (Table 3)

**Table 3:** Summary of health-related quality of life and subjective global assessment.

Variables	Baseline	Week 4	Week 8	Week 12	Week 16	Week 24	F	*P *value	Post hoc Comparison
	Mean (SD)	Mean (SD)	Mean (SD)	Mean (SD)	Mean (SD)	Mean (SD)			
Physical health components	58.2 (13.2)	74.5 (24.5)	75.5 (25.2)	65.3 (24.8)	67.0 (24.3)	69.5 (23.9)	3.998	0.098	
Mental health components	85.0 (34.2)	102.5 (42.2)	103.3 (42.6)	95.5 (39.0)	95.0 (39.1)	96.0 (37.7)	8.049	0.012*	Baseline < Week 4 and 8 Week 8 > Week 12, 16 and 24
Burden of kidney disease	61.3 (10.8)	70.0 (11.2)	72.5 (8.5)	72.0 (9.1)	76.5 (10.0)	78.5 (8.5)	17.913	0.010*	Baseline < Week 4, 8, 12, 16 and 24 Week 12 < Week 16 and 24
Symptoms and problems	107.7 (7.8)	113.8 (6.5)	116.7 (5.3)	109.5 (5.0)	109.8 (5.7)	110.5 (5.0)	16.126	0.000***	Week 8 > Week 4 > Baseline, week 12, 16 and 24
Effects of kidney disease on daily life	97.9 (13.2)	104.0 (10.5)	104.9 (10.4)	102.9 (9.5)	102.75 (11.31)	102.00 (10.25)	3.809	0.051	
SGA	6.4 (0.6)	6.6 (0.50)	6.5 (0.7)	6.5 (0.5)	6.7 (0.5)	6.8 (0.4)	2.969	0.015*	Baseline, Week 8 < Week 24 Week 12 < Week 16 and 24

*SGA* subjective global assessment.

*P < 0.05, **P < 0.01, ***P < 0.001.

The baseline scores of KDQOL-36™ are listed in Table 3. The scores of physical health components were similar throughout the 24 weeks. At week 4, the scores of mental health components, burden of kidney disease, symptoms and problems, and effects of kidney disease on daily life improved significantly. At week 8, only the score of symptoms and problems kept improving. But at week 12, the scores of mental health components, and symptoms and problems decreased significantly. At the end of study, the mental health components and burden of kidney disease showed improvement compared with baseline measurements significantly (*P* = 0.012, and *P* = 0.010, respectively).

The baseline SGA was 6.4 ± 0.6, which indicated normal nutritional status. Throughout the study, the SGA increased gradually, and the nutritional status was normal. At the end of the study, the SGA had increased 0.4 compared with the baseline (*P* = 0.015).

#### Laboratory data (Table 4)

**Table 4:** Summary of laboratory data.

Variables	Baseline	Week 4	Week 8	Week 12	Week 16	Week 24	F	*P *value	Post hoc Comparison
	Mean (SD)	Mean (SD)	Mean (SD)	Mean (SD)	Mean (SD)	Mean (SD)			
WBC (/μL)	6348 (2066.8)	6063.6 (2279.6)	6247.6 (1785.1)	6193.3 (1798.3)	6212.8 (2086.1)	6026.00 (1784.9)	0.467	0.745	
Hb (g/dL)	11.0 (1.2)	11.0 (1.2)	11.0 (1.3)	10.9 (1.2)	11.2 (1.1)	11.1 (1.1)	0.579	0.659	
Platelet (K/μL)	187.3 (82.4)	182.4 (79.)	194.2 (81.5)	194.0 (85.2)	180.8 (72.2)	185.8 (78.5)	1.557	0.201	
Glucose (mg/dL)	116.1 (43.7)	124.3 (43.9)	115.7 (41.1)	115.0 (38.2)	115.7 (43.3)	109.2 (42.3)	1.013	0.413	
Albumin (g/dL)	4.13 (0.28)	4.160 (0.318)	4.14 (0.29)	4.06 (0.29)	4.10 (0.28)	4.17 (0.36)	3.809	0.051	
BUN (mg/dL)	80.8 (18.8)	81.3 (20.7)	81.8 (16.8)	80.1 (11.7)	83.3 (16.3)	89.5 (17.6)	4.126	0.002**	Week 24 > Baseline, week 4, 8, 12, and 16
Creatinine (mg/dL)	12.2 (2.9)	12.7 (3.2)	12.3 (3.2)	12.0 (2.9)	11.6 (2.7)	11.8 (2.9)	7.366	0.000***	Week 16 = Week 24 < Baseline, week 4, 8, and 12
Uric acid (mg/dL)	8.1 (1.8)	7.9 (2.0)	8.0 (1.5)	8.3 (1.4)	8.1 (1.5)	8.3 (1.7)	0.741	0.453	
Na (mmol/L)	137.0 (3.2)	137.2 (3.4)	137.5 (2.9)	137.4 (3.0)	136.6 (3.3)	136.5 (2.8)	3.254	0.009**	Week 8 = Week 12 > Week 16 = Week 24
K (mmol/L)	4.8 (0.4)	4.8 (0.6)	4.9 (0.6)	4.7 (0.6)	4.8 (0.5)	5.1 (0.6)	4.684	0.001**	Week 24 > Baseline, week 4, 8, 12, and 16
Ca (mmol/L)	9.6 (0.6)	9.5 (0.6)	9.5 (0.6)	9.5 (0.7)	9.7 (0.7)	9.6 (0.6)	1.227	0.301	
P (mg/dL)	5.1 (1.5)	5.3 (1.3)	5.3 (1.4)	5.0 (1.5)	4.9 (1.3)	5.5 (1.5)	1.412	0.225	

*BUN* blood urea nitrogen, *Ca* calcium, *Hb* hemoglobin, *K* potassium, *Na* sodium, *P* phosphorus, *WBC* white blood cell.

*P < 0.05, **P < 0.01, ***P < 0.001.

During the study period, white blood count, hemoglobin and platelet count did not alter significantly. Serum glucose, albumin, uric acid, calcium, and phosphate levels fluctuated over time. At week 4, serum creatinine increased. At week 8, sodium reached the top. At the end of the study, the blood urea nitrogen (BUN) and potassium were elevated compared with the baseline (*P* < 0.002, and *P* = 0.001, respectively), possibly indicating more protein and potassium intake.

### Spearman correlation

By Spearman correlation, the monthly step count moderately correlated positively with 6MWT (Spearman correlation coefficient Rs = 0.472, *P* = 0.017) (Table 5). The monthly step count of HD patients was directly proportional to the 6MWT distance. The monthly step count was not correlated with STS-10, STS-60 or handgrip measurements. STS-10 was highly correlated negatively with STS-60 (Rs = − 0.703, *P* < 0.01), and moderately correlated negatively with 6MWT (Rs = − 0.458, *P* = 0.021) and handgrip measurement.

**Table 5:** Spearman correlation of steps per month and physical function.

	Steps per month	STS-10	STS-60	6MWT	HGS, non-dominant hand	HGS, dominant hand
Steps per month	1					
STS-10	− 0.240	1				
STS-60	0.396	− 0.703**	1			
6MWT	0.472*	− 0.458*	0.534**	1		
HGS, non-dominant hand	0.187	− 0.535*	0.622**	0.563**	1	
HGS, dominant hand	0.192	− 0.478*	0.567**	0.512**	0.958**	1

*STS-10* sit-to-stand 10, *STS-60* sit-to-stand 60, *6MWT* 6-m walk test, *HGS* hand grip strength.

*P < 0.05, **P < 0.01.

The monthly step count also moderately correlated positively with SGA (Rs = 0.438, *P* = 0.029) (Table 6); the higher the monthly step count, the higher the SGA was found, which indicated better nutritional status. The monthly step count was not correlated with HRQoL (Table 7) or laboratory data. The physical health components were strongly correlated negatively with burden of kidney disease (Rs = − 1.000, *P* < 0.01).

**Table 6:** Spearman correlation of steps per month and subjective global assessment.

	Steps per month	SGA
Steps per month	1	
SGA	0.438*	1

*SGA* subjective global assessment.

*P < 0.05, **P < 0.01.

**Table 7:** Spearman correlation of steps per month and health-related quality of life.

	Steps per month	Physical health components	Mental health components	Burden of kidney disease	Symptoms and problems	Effects of kidney disease on daily life
Steps per month	1					
Physical health components	− 0.371	1				
Mental health components	0.294	− 0.647	1			
Burden of kidney disease	0.000	− 1.000**	0.738	1		
Symptoms and problems	− 0.249	0.714	− 0.559	− 0.400	1	
Effects of kidney disease on daily life	− 0.190	− 0.314	0.412	− 0.400	0.262	1

*P < 0.05, **P < 0.01.

## Discussion

In this study, the physical activity of 25 stable HD patients improved significantly after a 24-week intervention using wearable devices, a health-management platform, and social media. By the end of the study, the daily step count increased by 1658 steps, the STS-10 had shortened by 4.4 s, the STS-60 increased 12 repetitions, and the 6MWT increased by 55.4 m compared with the baseline. The metal health components and burden of kidney disease of HRQoL and nutritional status, measured by KDQOL-36™ and SGA, respectively, also improved significantly. By Spearman correlation, the monthly step count was correlated positively with the 6MWT distance and SGA score; the higher the monthly step count, the longer the 6MWT distance and higher SGA score were noted.

In one Spanish prospective study to determine the reliability of outcomes of physical performance tests for HD patients showed high intraclass correlation coefficients (≥ 0.88) for STS-10, STS-60, the one-leg heel-rise test, and the HGS test, suggesting good relative reliability^23^. The minimal detectable change scores at 90% confidence intervals (CI) were as follows: 8.4 s for the STS-10, 4 repetitions for the STS-60, 66.3 m for the 6MWT, 3.4 kg for HGS. Those degree of change was necessary to discriminate between the true effects of exercise interventions and the inherent variability of the cohort. In our study, only the improvement of STS-60 (increase 12 repetitions) reached the minimal detectable change score. It meant the change of STS-60 was due to an intervention. There were no relevant changes in HGS after our intervention. Some studies had similar results^32^. The exercise training was less focused on the upper limbs may explain these findings.

In the assessment of HRQoL, significant improvement of the mental components and burden of kidney disease were observed. The participants felt less depressed, less frustrated, and they were not the burdens of family. Unlike the secondary analysis of the EXCITE trial^33^, which revealed cognitive function in the Kidney Disease Quality of Life- Short Form significantly reduced in patients of the control arm, while it remained substantially unchanged in those of the active arm. Because the tools in evaluation of HRQoL were different, the beneficial effects of exercise were diverse.

### Types and doses of physical activity

A systematic review and meta-analysis of exercise interventions for ESRD patients on HD or peritoneal dialysis (from 27 studies with 1156 participants) showed that exercise, regardless of modality, improved objective measures of physical activity, such as increased 6MWT distance, sit-to-stand time or repetitions, and grip strength as well as step and stair climb times or repetitions, dynamic mobility, and short physical performance battery scores^12^. Another systematic review with network meta-analysis showed that the combined training (aerobic plus resistance training) was the most effective modality for increasing aerobic capacity and blood pressure control in HD patients^34^. Compared with data from one of the largest randomized control trails in the area with 296 participants, the exercise group experienced a 6-month personalized walking exercise program and demonstrated an improvement of 39 m in 6MWT and HRQoL in subscales of cognitive function and social interaction^35^. Our intervention of wearable device-based walking comprised aerobic exercise, and the calisthenics included stretching, aerobic and resistance training. These home-based exercises could improve cardiopulmonary endurance and flexibility, and achieve long-term effects in a safe way.

A daily step count goal of 7500 steps was set according to a prospective cohort study of 18,289 women in the United States which displayed survival benefits^36^. A prospective Japanese cohort study found a substantial mortality benefit among the disability-free HD patients who engaged in at least 4000 steps per day^10^. This provided a basis for a minimum initial recommendation that kidney health providers could provide for stable HD patients. Another retrospective cohort study was performed in 192 Japanese HD patients with a 7-year follow-up; a mortality of 20.8% was noted^6^. The average number of steps taken per non-dialysis day was used as a measure of physical activity (about 4421 ± 3048 steps). The patients were divided into three categories according to the percentage changes in physical activity between baseline and 12 months. The hazard ratio on multivariate analysis in patients with decreased physical activity (> 30% decrease) was 3.68 (95% CI 1.55–8.78; *P* = 0.01) compared to those with increased physical activity (> 30% increase). Reductions in physical activity were significantly associated with poor prognosis and were independent of patient characteristics and baseline physical activity. Therefore, improved prognosis in HD patients required the means of preventing a decline in physical activity over time. Compared with this pedometer-guided exercise study, the daily step count tracked by our participants exceeded expectations, from an average 5469 steps per day at baseline to 7127 steps per day by the end of the study. The daily step count did not exceed that of the general population and may be related to inactivity during HD therapy. The trend of increased step count suggested the effectiveness of this new model of telehealth.

The 2015 Taiwan Chronic Kidney Disease Clinical Guidelines^37^ encourage physical activity compatible with cardiovascular health and tolerance, and aim for at least 30 min activity five times per week. The NICE clinical practice guidelines about exercise and lifestyle emphasize physical activity and exercise in the HD population where there are no contraindications^11^. HD patients were recommended to aim for 150 min of moderate intensity activity or 75 min of vigorous activity a week, or a mixture of both as per the UK Chief Medical Officers’ Guidelines. This may include a combination of interdialytic and intradialytic exercise^38^. The best protocol for CKD patients including maintenance HD patients remains to be established^39^. Most studies focused on intradialytic exercise, and some studies included independent home exercise^32,33,38^. Wearable devices were used along with a remote-control model via a health management platform. The exercise program with only walking and calisthenics at least three sessions per week appeared easy to implement in clinical practice with high attendance among participants.

### Design of the telehealth from digital health interventions (DHIs)

As in our previous randomized controlled study, wearable devices were applied, with a health management platform, and social media support for 12 weeks to people diagnosed with CKD stage 1–4. The intervention group gained significantly higher scores for self-efficacy and self-management. HRQoL and the daily step counts increased in the intervention group. The decline in eGFR were significantly slower in the intervention group (− 0.56 vs. − 4.58 mL/min/1.73 m^2^). These results outlined a new self-management model to promote healthy lifestyle behaviors in CKD patients^17^. In the present investigation, a longer-term intervention was performed to maintenance HD patients, and it was documented that their physical function and HRQoL improved. A randomized control trial was not performed because all participants should be encouraged to pursue healthy habits. This study also provided a new model for promoting sustainable healthy lifestyles in stable HD patients.

In a cross-sectional observational study, 44% of HD patients did not believe they had an important role in managing their health. The average patient activation-score by Patient Activation Measure-13 was 51 (0–100), which was lower than other chronic patient groups. Multiple linear regression showed that older patients, who reported being in bad health, treated in a particular hospital, without leisure-time activities, and living in a residential care home, had a lower patient activation level^40^. Our participants were younger than the general HD population. They were more motivated to increase physical activity and strengthen self-management. As the mean age of people undergoing dialysis therapy has increased, the importance of maintaining the functional capacity and increasing physical activity should be emphasized.

DHIs were used in the HD field mostly for dialysis service management, dialysis procedure, anemia management, and arteriovenous fistula assessment^41^. This interventional study is unique in the combination of a wearable device, a health management platform, and social media in a population under regular HD. Few studies have adopted wearable devices, a health management platform, and social media to quantify motor performance with immediate feedback to empower participants^42^. Suggestions were provided to the participants according to uploaded images of their diet, so they could adjust their diet. Our intervention type is a multifactorial behavior modification which includes exercise and diet. During a global pandemic such as coronavirus (COVID-19), telemedicine is a trustworthy method of clinical practice to limit travel and exposure and encourage social distancing while inspiring care from healthcare providers. The use of new technology is a practical way to deliver health care and increase interactions between medical staff and HD patients. However, it requires technological literacy among the patient population. More investigations are needed into the potential effects of DHIs on health promotion and lifestyle in HD patients, and even in patients receiving peritoneal dialysis or post-renal transplantation.

## Limitations

Participants in this study were recruited from a teaching hospital and a HD center of central Taiwan. Participants were younger than the general HD population in Taiwan, so the results could not be used among the general HD population. The sample was relatively small due to a lack of motivation among participants. The small sample size may have amplified the differences before and after the intervention. Although physical activity and HRQoL outcomes improved after the 24-week intervention, the long-term effects of the intervention should also be further evaluated. A follow-up period of at least 1–3 years would allow for better evaluation of the long-term effectiveness, such as cardiovascular outcome and mortality. No control group was included in this analysis, making it impossible to understand whether the improvements were due to the intervention or not. Finally, our intervention design meant that we could not distinguish between the effects of exercise, dietary intervention, or emotional support via social media.

## Conclusion

DHIs that combined wearable devices, a health management platform, and social media could strengthen physical activity and improve the HRQoL and nutrition of maintenance HD patients. The results outline a new model that can promote healthy lifestyle behaviors as part of routine care in HD patients.

## Data Availability

Original data will be available upon request from author: Wen-Yi Li (E-mail: b5401068@gmail.com).
